# Two amino acid residues in the N-terminal region of the polymerase acidic protein determine the virulence of Eurasian avian-like H1N1 swine influenza viruses in mice

**DOI:** 10.1128/jvi.01293-24

**Published:** 2024-08-30

**Authors:** Yuying Yang, Chengzhi Xu, Naixin Zhang, Yunfei Wan, Yunpu Wu, Fei Meng, Yan Chen, Huanliang Yang, Liling Liu, Chuanling Qiao, Hualan Chen

**Affiliations:** 1 State Key Laboratory for Animal Disease Control and Prevention, Harbin Veterinary Research Institute, Chinese Academic Agricultural Sciences, Harbin, China; University Medical Center Freiburg, Freiburg, Germany

**Keywords:** mechanism, PA protein, rEA H1N1, swine influenza virus, virulence

## Abstract

Reassortant Eurasian avian-like H1N1 (rEA H1N1) viruses carrying the internal genes of H1N1/2009 virus have been circulating in pigs for more than 10 years and have caused sporadic human infections. The enhanced virulence phenotype of the rEA H1N1 viruses highlights potential risks to public health. However, the molecular mechanism underlying the viral pathogenicity of the currently circulating rEA H1N1 viruses remains unclear. In this study, we found that two naturally isolated rEA H1N1 swine influenza viruses, A/swine/Liaoning/FX38/2017 (FX38) and A/swine/Liaoning/SY72/2018 (SY72), possessed similar genetic characteristics but exhibited significantly different pathogenicity in a mouse model. Using reverse genetics, we demonstrated that amino acid mutations at positions 100 and 122 in the polymerase acidic (PA) protein had individual and synergistic effects on the polymerase activity and viral replication capacity *in vitro*, as well as the viral pathogenicity in mice. Furthermore, we revealed that amino acid residue 100 in PA influenced the transcription of viral RNA (vRNA) by altering the endonuclease activity, and amino acid residue 122 affected the synthesis of complementary RNA and messenger RNA by altering the RNA-binding ability and endonuclease activity of the PA protein. Taken together, we identified that two naturally occurring amino acid mutations in PA derived from H1N1/2009 virus are crucial determinants of the virulence of rEA H1N1 viruses and revealed the differential mechanism by which these two mutations affect the transcription and replication of vRNA. These findings will extend our understanding of the roles of PA in the virulence of influenza A viruses.

**IMPORTANCE:**

Multiple genetic determinants are involved in the virulence of influenza A viruses. In this study, we identified two naturally occurring amino acid mutations, located at residues 100 and 122 in the polymerase acidic (PA) protein, which are associated with viral polymerase activity, replication competence, and pathogenicity in mice. In particular, we clarified the specific mechanism by which the two residues play an important role in viral transcription and replication. These findings will help to improve understanding the functions of amino acid residues in the N-terminal region of the PA protein involved in the pathogenicity of influenza A viruses.

## INTRODUCTION

Influenza A viruses are enveloped negative-strand RNA viruses with genomes composed of eight viral RNA (vRNA) segments that encode at least 10 proteins, which include polymerase basic protein 2 (PB2), polymerase basic protein 1 (PB1), polymerase acidic protein (PA), hemagglutinin (HA), nucleoprotein (NP), neuraminidase (NA), matrix proteins (M1 and M2), and nonstructural proteins (NS1 and NS2). Virus subtypes are classified by different combinations of the HA and NA. H1N1 is the main subtype co-circulating in avian, swine, and human populations (1). The transmission of H1N1 from birds to pigs was first reported in 1979 in Europe and soon afterward in Asia (2–4) and therefore was referred to as Eurasian avian-like H1N1 swine influenza virus (EA H1N1 SIV). Emergence of H1N1/2009 virus in humans highlights the important role of pigs in the recombination and cross-host transmission of influenza viruses (5). Several studies have demonstrated that the internal genes of H1N1/2009 viruses had good genetic compatibility with the other surface genes under experimental and natural conditions, which usually enhanced the virulence and transmission ability in mammals (6–10). Another concern about the H1N1/2009 virus is that the variant H3N2 viruses carrying the M genes of H1N1/2009 viruses have caused human infections in a certain region of the USA (11, 12).

The pathogenicity of influenza A viruses is determined by multiple factors, including one or more amino acid differences of a single gene fragment or the synergistic action of multiple gene fragments (13–20). Moreover, the gradual acquisition of amino acid changes in viral proteins (i.e., polymerase proteins, HA, and NP) are important for host restriction (21–25). In recent years, reassortant EA H1N1 (rEA H1N1) viruses, bearing the HA and NA genes of EA H1N1 viruses, PB2, PB1, PA, and NP genes of H1N1/2009 viruses, M gene of EA H1N1 or H1N1/2009 virus, and NS gene of triple reassortant H1N2 virus, have become the predominant strains in pig populations and caused sporadic human infections in China, which highlights the potential threats to public health (26–30).

Mice, guinea pigs, and ferrets are widely used as the mammalian models to evaluate the pathogenesis and transmission of influenza viruses (31, 32). In this study, we investigated the genetic basis of two rEA H1N1 SIVs—A/swine/Liaoning/FX38/2017 (FX38) and A/swine/Liaoning/SY72/2018 (SY72)—that are genetically similar but differ in virulence in BALB/c mice. We identified the key molecular determinants involved in their virulence differences and also revealed the underlying mechanisms.

## RESULTS

### FX38 and SY72 viruses possessed similar genetic characteristics but exhibited significantly different virulence in mice

**Fig 1:**
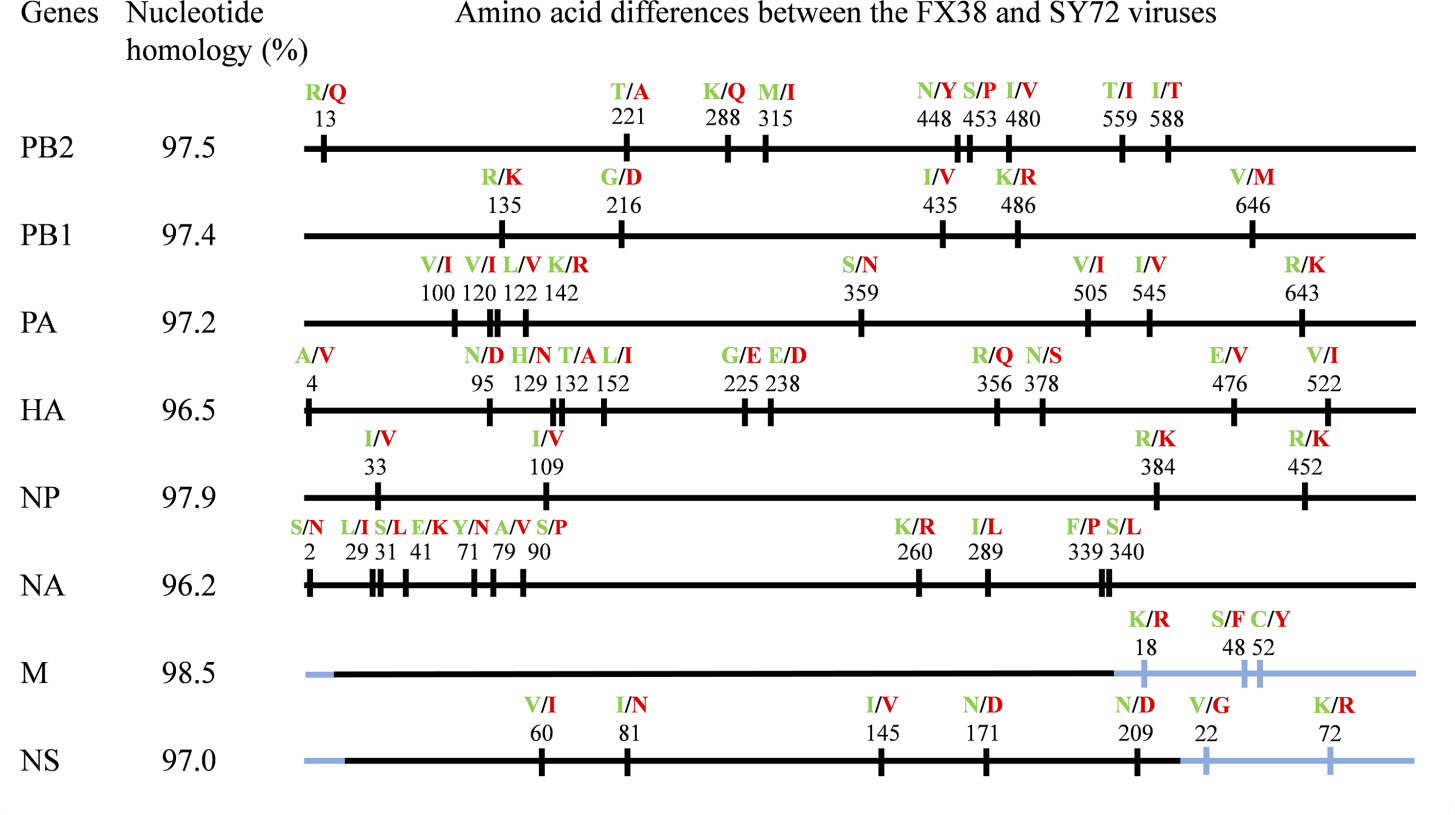
Genetic homology analysis and amino acid differences between the FX38 and SY72 viruses. The viruses were isolated during routine surveillance, and virus stocks were propagated in 10-day-old specific pathogen-free embryonated chicken eggs. The eight gene segments of the viruses were fully sequenced and aligned by Clustal W, using Lasergene 7.1. The different amino acids are shown as single letters at the indicated positions. Amino acids of the FX38 virus are shown in green, and those of the SY72 virus are shown in red. The amino acid positions of HA are H3 numbering. No amino acid difference was observed between the M1 protein of these two viruses. The M2 and NS2 proteins are shown in blue.

**Fig 2:**
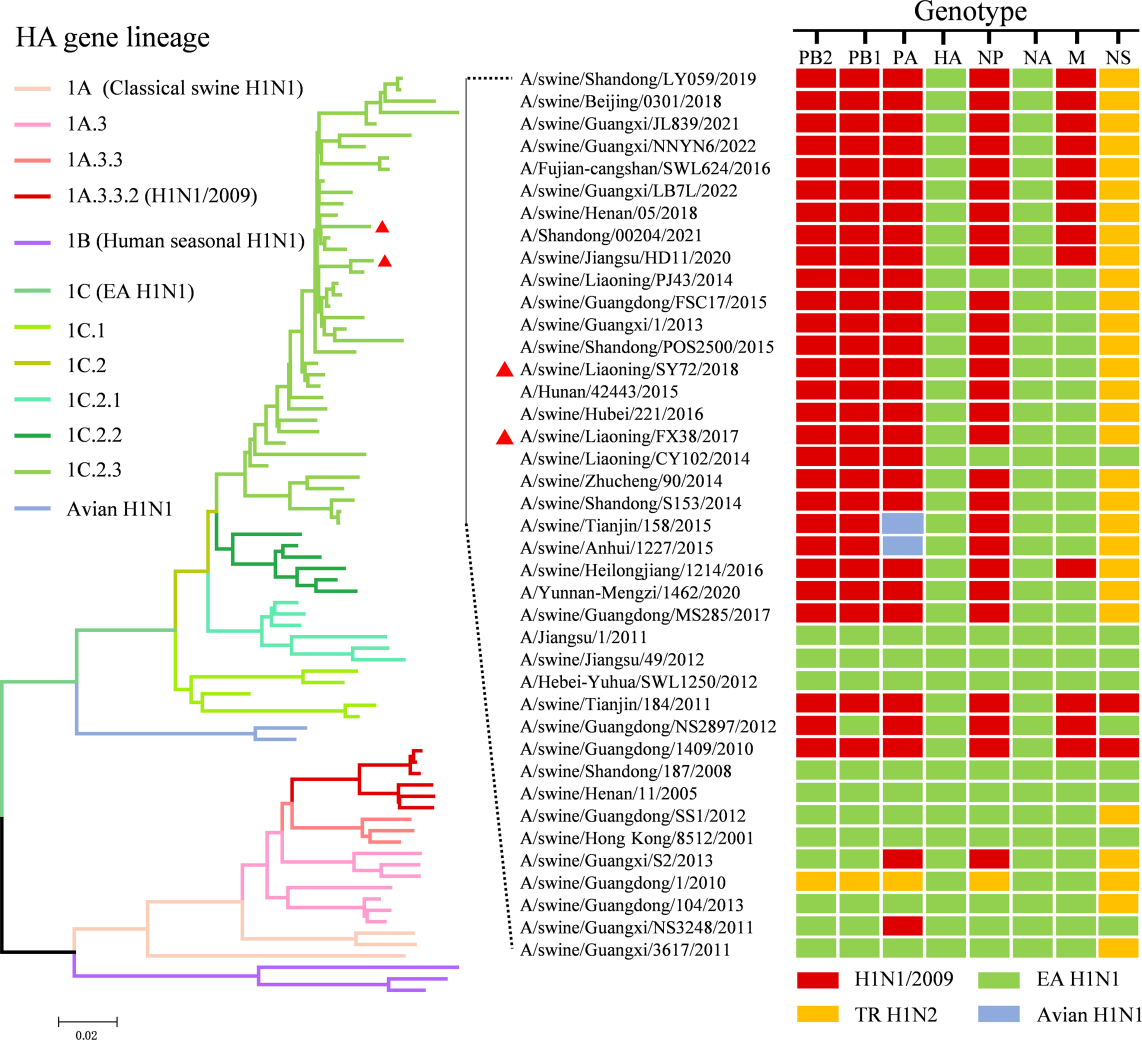
Phylogenetic analyses of the FX38 and SY72 viruses. HA gene tree was constructed with MEGA (v7.0.26) using the neighbor-joining method with 1,000 bootstrap replicates, based on nucleotide positions 33 to 1,733. The colors of the bars represent the clades in the tree. The red triangles indicate viruses characterized in this study. Clade numbers are indicated on the left of the panel. Colored boxes show the lineage classification of each gene segment of viruses belonging to the 1C.2.3 subclade. EA, Eurasian avian-like; TR, triple reassortant.

To understand the genetic relationships of the FX38 and SY72 viruses, these two viruses were sequenced and phylogenetically analyzed. As shown in Fig. 1, sequence alignment of the eight gene segments showed that these two viruses shared 96.2% to 98.5% identity at the nucleotide level. There were 58 different amino acids between the FX38 and SY72 viruses, which were mapped in the PB2, PB1, PA, HA, NP, NA, M2, NS1, and NS2 proteins, respectively (Fig. 1). We constructed an unrooted phylogenetic tree using the HA genes of the FX38 and SY72 viruses and 79 H1N1 reference strains, and found that these two viruses fell into the 1C.2.3 subclade, including EA H1N1 viruses previously isolated in pigs and humans. Phylogenetic analysis of the other seven genes showed that the NA and M genes of these two viruses were derived from EA H1N1 viruses, the PB2, PB1, PA, and NP genes from H1N1/2009 viruses, and the NS genes from triple-reassortant H1N2 viruses. Furthermore, we analyzed the genomic constellation of the H1N1 viruses bearing the 1C.2.3 subclade HA and found that there were at least 12 genotypes of EA H1N1 viruses isolated from pigs in China since 2001 (Fig. 2).

**Fig 3:**
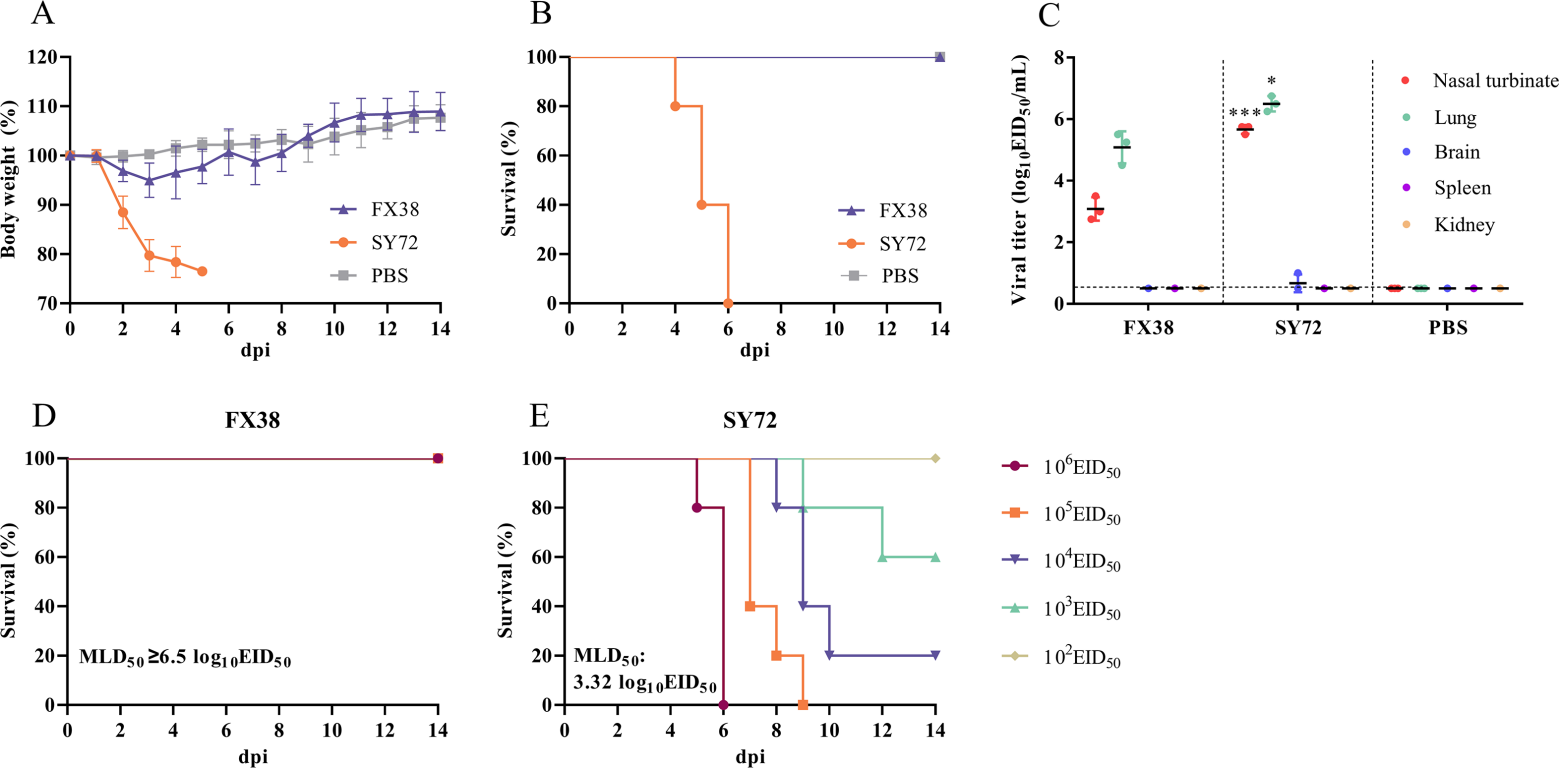
Virulence of the FX38 and SY72 viruses in mice. (**A**) Body weight, (**B**) survival, and (**C**) virus titers in the organs of mice infected with the indicated viruses. Groups of eight mice were intranasally inoculated with 10^6^ EID_50_ (each in 50 µL) of the indicated viruses. Body weight and survival were monitored daily for 14 days after infection. Mice that lost ≥25% of baseline body weight were euthanized. The values represent the average (± SD) body weight of five mice compared to baseline. At 3 dpi, the organs (brain, nasal turbinate, lungs, spleen, and kidneys) of three mice were collected and titrated in eggs. A value of 0.5 was assigned to virus titration-negative samples for statistical purposes. Each dot represents the viral titer of each mouse, and the horizontal bars indicate the mean virus titers of three mice. Asterisks indicate virus titers significantly different from those of the FX38 virus (*, *P* < 0.05; ***, *P* < 0.001). (**D and E**) MLD_50_ values of the FX38 and SY72 viruses. Five mice per group were intranasally inoculated with 10^2^, 10^3^, 10^4^, 10^5^, or 10^6^ EID_50_ (each in 50 µL) of the indicated viruses. The MLD_50_ values were calculated by the Reed–Muench method (33) and expressed as log_10_ EID_50_.

To evaluate the virulence of the FX38 and SY72 viruses, groups of eight BALB/c female mice were intranasally inoculated with 10^6^ EID_50_ of the virus. As shown in Fig. 3A and B, all mice infected with the FX38 virus showed slight body weight loss within 7 days post-infection (dpi), whereas the mice infected with the SY72 virus showed weight loss with the ratio exceeding 25% and were, therefore, considered dead. Viral replication in organs (including the brain, nasal turbinate, lungs, spleen, and kidneys) of the two viruses were determined at 3 dpi and shown in Fig. 3C. The viral titers detected in the nasal turbinates and lungs of mice inoculated with the FX38 virus were 3.1 and 5.1 log_10_ EID_50_/mL, respectively. The viral titers detected in the nasal turbinates and lungs of mice infected with the SY72 virus were significantly higher than those of mice infected with the FX38 virus, with the titers of 5.7 and 6.5 log_10_ EID_50_/mL, respectively. Notably, no virus was detected in the other organs of mice infected with the two viruses, with the exception that the SY72 virus was titrated in the brain of one mouse. Furthermore, we determined the 50% mouse lethal dose (MLD_50_) values of these two viruses by inoculating groups of five mice with 10^2^–10^6^ EID_50_ of the FX38 and SY72 viruses and found that the MLD_50_ value of the FX38 virus was ≥6.5 log_10_ EID_50_ (Fig. 3D), while the MLD_50_ value of the SY72 virus was 3.32 log_10_ EID_50_ (Fig. 3E).

### PA gene is the key factor determining the viral replication capacity and polymerase activity

**Fig 4:**
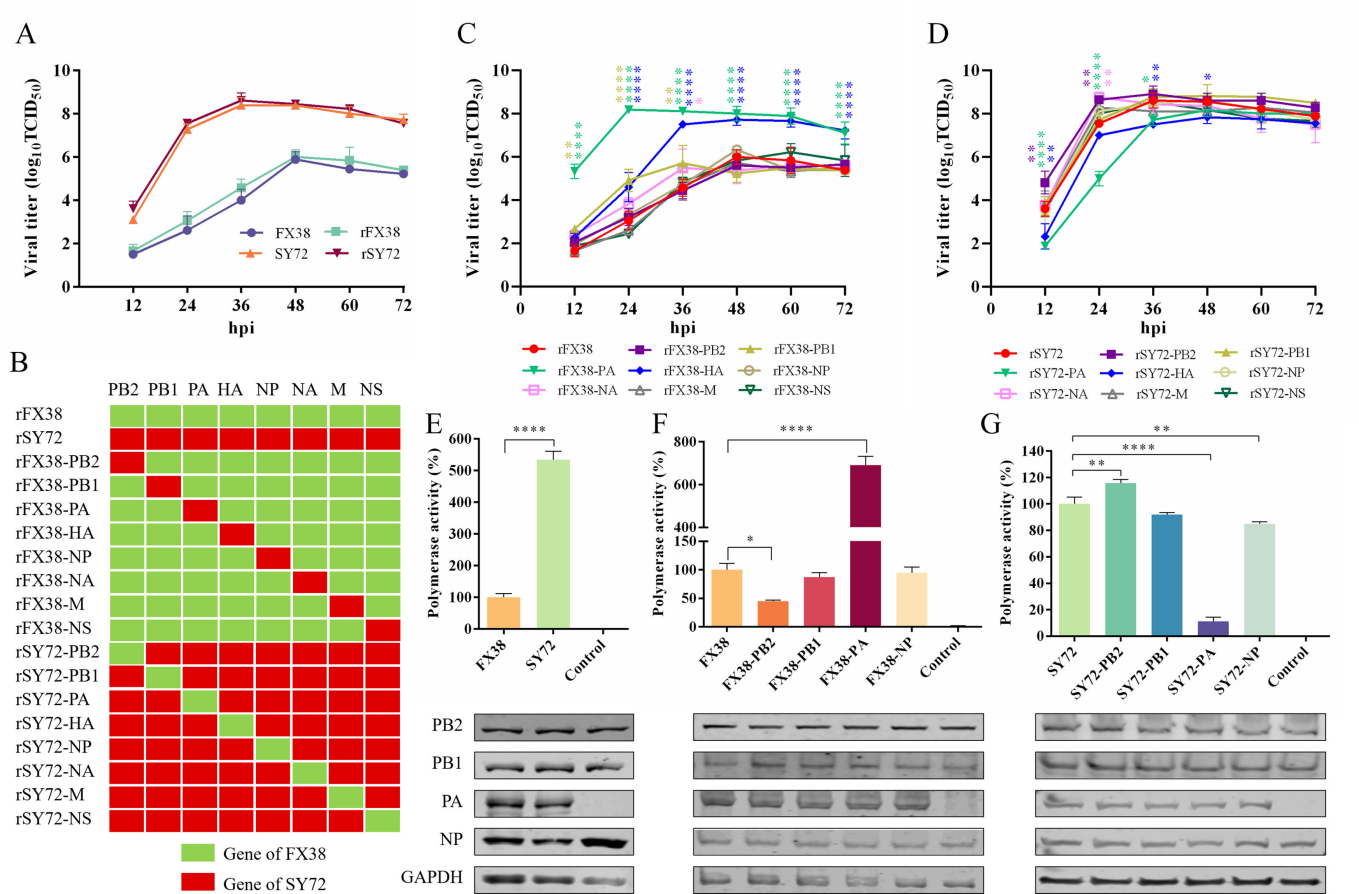
Determination of the gene fragments influencing viral replication capacity and polymerase activity. (**A**) Replication capacities of the wild-type and rescued parental rFX38 and rSY72 viruses *in vitro*. MDCK cells were infected with the indicated viruses at a multiplicity of infection (MOI) of 0.001. Supernatants were collected at 12-h intervals until 72 hpi and titrated in MDCK cells by 50% tissue culture infectious dose assay. (**B**) The scheme of 16 reassortant viruses by exchanging a single-gene fragment in the rFX38 and rSY72 backgrounds. (**C and D**) Replication capacities of the single-gene-substitution reassortant viruses in the background of FX38 and SY72. (**E**) Polymerase activities of the wild-type vRNP of the FX38 and SY72 viruses. The polymerase activity of the wild-type vRNP of FX38 was set at 100%. (**F and G**) Polymerase activities of the vRNP reconstituted by single-gene substitution in the background of FX38 and SY72 viruses, respectively. HEK293T cells were transfected with PB2, PB1, NP, and PA protein expression plasmids, together with a minigenome encoding the luciferase gene (pPolI-Luc) and an internal control plasmid encoding *Renilla* luciferase (pRL-TK). HEK293T cells transfected with the plasmid mixture without PA gene were used as a negative control. The polymerase activity was normalized to that of the wild-type vRNP of FX38 or SY72. The data are presented as the means (± SD) of three independent experiments. *P <0.05; **P < 0.01; ****P < 0.0001. The cell lysates were also analyzed for expression of the three polymerases and NP proteins using western blot.

To explore the molecular basis for these differences in virulence, we rescued the recombinant viruses, which were designated rFX38 and rSY72, and compared their replication capacities with the wild-type viruses in MDCK cells. As shown in Fig. 4A, the rFX38 and rSY72 viruses shared the same replication characteristics as the wild-type FX38 and SY72 viruses, respectively, *in vitro*. Then, we generated 16 reassortant viruses by exchanging a single gene fragment in the rFX38 and rSY72 backgrounds according to the scheme shown in Fig. 4B and tested their growth dynamics in MDCK cells. Compared to the rFX38 virus, the rFX38-PA virus exhibited significantly increased replication capacity during 12–72 hours post-infection (hpi) (Fig. 4C). Furthermore, replication titers of the rFX38-HA and rFX38-PB1 viruses were enhanced at 24–72 and 12–36 hpi, respectively (Fig. 4C). The titers of the rSY72-PA and rSY72-HA viruses were significantly lower than that of the rSY72 virus at 12–36 and 12–48 hpi, respectively (Fig. 4D). Interestingly, the replication titers of the rSY72-PB2 virus were higher than that of the rSY72 virus at 12 and 24 hpi, respectively (Fig. 4D).

Next, we determined the polymerase activities of the wild-type viral ribonucleoprotein (vRNP) complexes of the FX38 and SY72 viruses in HEK293T cells and found that the polymerase activity of the SY72 vRNP was 5.3-fold higher than that of the FX38 vRNP (Fig. 4E). To investigate which gene(s) had effects on the polymerase activities of the vRNP complexes, we reconstituted the different vRNP by replacing a single PB2, PB1, PA, or NP gene using the vRNP of FX38 and SY72 as backgrounds. As shown in Fig. 4F, the polymerase activity of the reconstituted vRNP containing PA gene of the SY72 virus was 6.9-fold higher than that of the FX38 vRNP. In contrast, the polymerase activity of the reconstituted vRNP containing PA gene of the FX38 virus was 8.9-fold lower than that of the SY72 vRNP (Fig. 4G). Interestingly, unlike the PA gene, the PB2 gene exhibited reverse effects on the polymerase activities of the reconstituted vRNP. The single-gene substitution of PB1 or NP had no effect on the polymerase activities of the reconstituted vRNP (Fig. 4F and G). These results indicate that the PA gene is a crucial factor determining the replication capacity and polymerase activity of rEA H1N1 SIV.

### Amino acid mutations at positions 100 and 122 in PA alter the viral replication capacity and polymerase activity

**Fig 5:**
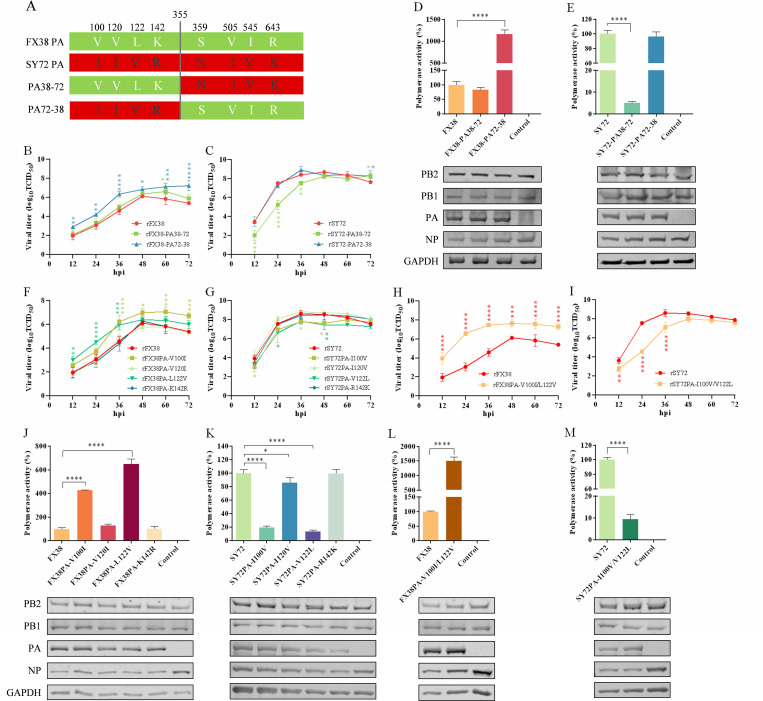
Determination of the amino acid residues in PA affecting viral replication capacity and polymerase activity. (**A**) The construction scheme of two chimeric PA. (**B and C**) Replication capacities of the chimeric reassortant viruses in the background of the FX38 and SY72 viruses in MDCK cells. (**D and E**) Polymerase activities of the reconstituted vRNP containing the chimeric PA in the backbone of the FX38 and SY72 viruses. (F–I) Replication capacities of the site-directed mutants in the background of the FX38 and SY72 viruses in MDCK cells. (J–M) Polymerase activities of the reconstituted vRNP containing the mutated PA in the backbone of the FX38 and SY72 viruses. Growth kinetics and minigenome assays were performed as described in the legend for Fig. 4. In each panel, the mean (± SD) of three replicates is shown. *P < 0.05; **P < 0.01; ***P < 0.001; ****P < 0.0001.

As shown in Fig. 1, the PA protein of the FX38 and SY72 viruses differed at eight amino acids (positions 100, 120, 122, 142, 359, 505, 545, and 643), of which four (positions 100, 120, 122, and 142) were located in the N-terminal region of the PA protein, and the other four were located in the C-terminal region. To identify the amino acid residue(s) that contributed to the differences in replication capacity of the two viruses, we rescued four viruses carrying the chimeric PA using the strategy shown in Fig. 5A. As shown in Fig. 5B, the replication titers of the rFX38-PA72-38 virus were significantly higher than those of the rFX38 virus, whereas the titers of the rFX38-PA38-72 and rFX38 viruses were comparable at all timepoints. Meanwhile, the replication titers of the rSY72-PA38-72 virus were significantly lower than those of the rSY72 virus at 12–36 hpi. However, the rSY72-PA72-38 virus showed similar replication capacities with the rSY72 virus at all timepoints (Fig. 5C). As shown in Fig. 5D, the polymerase activity of the reconstituted vRNP containing PA72-38 was 11.7-fold higher than of the FX38 vRNP, whereas the polymerase activity of the reconstituted vRNP containing PA38-72 was comparable with that of the FX38 vRNP. Meanwhile, the polymerase activity of the reconstituted vRNP containing PA38-72 was 19.5-fold lower than that of the SY72 vRNP, whereas the polymerase activity of the reconstituted vRNP containing PA72-38 was similar with that of the SY72 vRNP (Fig. 5E).

To pinpoint the amino acids influencing viral replication capacity, four mutants bearing the individual amino acid substitution at positions 100, 120, 122, and 142 in PA were rescued, and their growth curves were tested. As shown in Fig. 5F, the rFX38PA-V100I and rFX38PA-L122V viruses showed stronger replication capacities than the rFX38 virus at 36–72 and 12–36 hpi, respectively. A single amino acid substitution at position 120 or 142 in PA had no effect on the viral replication capacity. However, the amino acid substitutions of I100V and V122L reduced the replication titers of the rSY72PA-I100V and rSY72PA-V122L viruses, respectively, in the early stage of infection (i.e., 12–48 hpi) (Fig. 5G). Furthermore, we rescued two reassortants containing a double amino acid mutation at positions 100 and 122 in PA and examined their replication capacities. The replication titers of the rFX38PA-V100I/L122V virus were significantly higher than those of the rFX38 virus at all timepoints (Fig. 5H). Meanwhile, the replication titers of the rSY72PA-I100V/V122L virus were significantly lower than those of the rSY72 virus at 12–36 hpi (Fig. 5I). Next, we further tested the polymerase activities of the mutant vRNPs by introducing the individual amino acid mutation into the PA gene of FX38 at positions 100, 120, 122, and 142, respectively. The polymerase activities of vRNP containing FX38PA-V100I and FX38PA-L122V were enhanced by 4.3- and 6.5-fold, respectively, compared with that of the FX38 vRNP. However, the polymerase activities of the vRNP containing FX38PA-V120I and FX38PA-K142R were comparable with that of the FX38 vRNP (Fig. 5J). When the reverse mutations at positions 100, 120, 122, and 142 were introduced into the PA gene of SY72, the polymerase activities of the vRNP containing SY72PA-I100V and SY72PA-V122L were reduced by 5.2- and 7.2-fold, respectively, compared with that of the SY72 vRNP. However, the polymerase activities of the vRNP containing SY72PA-I120V and SY72PA-R142K were comparable with that of the SY72 vRNP (Fig. 5K). Of note, when the two mutations (V100I and L122V) were simultaneously introduced into the PA gene of FX38, the polymerase activity of the vRNP containing FX38PA-V100I/L122V was enhanced by 15-fold compared with that of the FX38 vRNP (Fig. 5L). Concurrently, the polymerase activity of the vRNP containing SY72PA-I100V/V122L was reduced by 10.7-fold compared with that of the SY72 vRNP (Fig. 5M). Taken together, these results indicate that a single mutation at position 100 or 122 in PA affects the viral replication capacity *in vitro* and polymerase activity in HEK293T cells. A double mutation at positions 100 and 122 has synergistic effects on the viral replication capacity and polymerase activity of rEA H1N1 SIV.

### Two amino acids at positions 100 and 122 in PA are crucial determinants of the viral pathogenicity in mice

**Fig 6:**
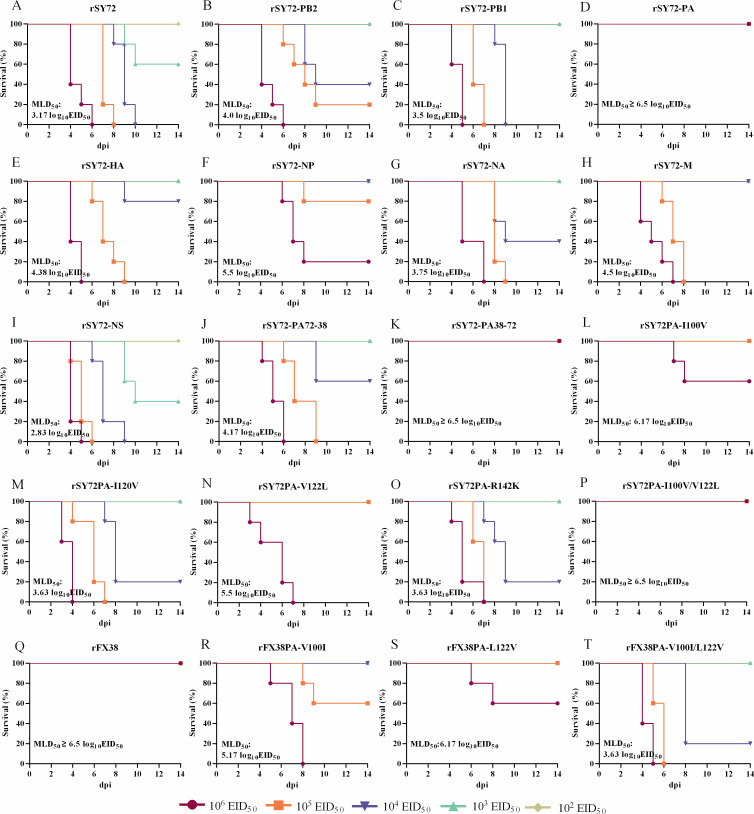
Effects of amino acid substitutions at positions 100 and 122 in the PA protein on pathogenicity of the FX38 and SY72 viruses in mice. (A–P) MLD_50_ values of the rSY72 virus and its single-gene reassortants, chimeric reassortants, and site-directed mutants. (Q–T) MLD_50_ values of the rFX38, rFX38-PA-V100I, rFX38-PA-L122V, and rFX38PA-V100I/L122V viruses. MLD_50_ determination was performed as described in the legend for Fig. 3.

To identify which gene(s) are responsible for the virulence differences of the FX38 and SY72 viruses in mice, we determined the MLD_50_ values of the rSY72 virus and its single-gene reassortants. The pathogenicity of the rSY72-PA, rSY72-HA, rSY72-NP, and rSY72-M viruses was attenuated, with the MLD_50_ values approximately 2,138-, 16-, 214-, and 21-fold higher than that of the rSY72 virus, respectively. The pathogenicity of the rSY72-PB2, rSY72-PB1, rSY72-NA, and rSY72-NS viruses was comparable with that of the rSY72 virus (Fig. 6A through I). We further determined the MLD_50_ values of the chimeric reassortants and site-directed mutants in the rSY72 background and found that the pathogenicity of the rSY72-PA38-72, rSY72PA-I100V, rSY72PA-V122L, and rSY72PA-I100V/V122L viruses was attenuated, with the MLD_50_ values approximately 2,138-, 1,000-, 214-, and 2,138-fold higher than that of the rSY72 virus, respectively (Fig. 6J through P), We also tested the MLD_50_ values of rFX38 and its site-directed mutants. The pathogenicity of the rFX38PA-V100I, rFX38PA-L122V, and rFX38PA-V100I/L122V viruses was increased, with the MLD_50_ values approximately 21-, 2-, and 741-fold lower than that of the rFX38 virus, respectively (Fig. 6Q through T). These results indicate that the amino acid residues 100 and 122 in PA individually and synergistically determine the viral pathogenicity of rEA H1N1 SIV in mice.

### Two amino acid mutations at positions 100 and 122 in PA affect the transcription and replication of vRNA

**Fig 7:**
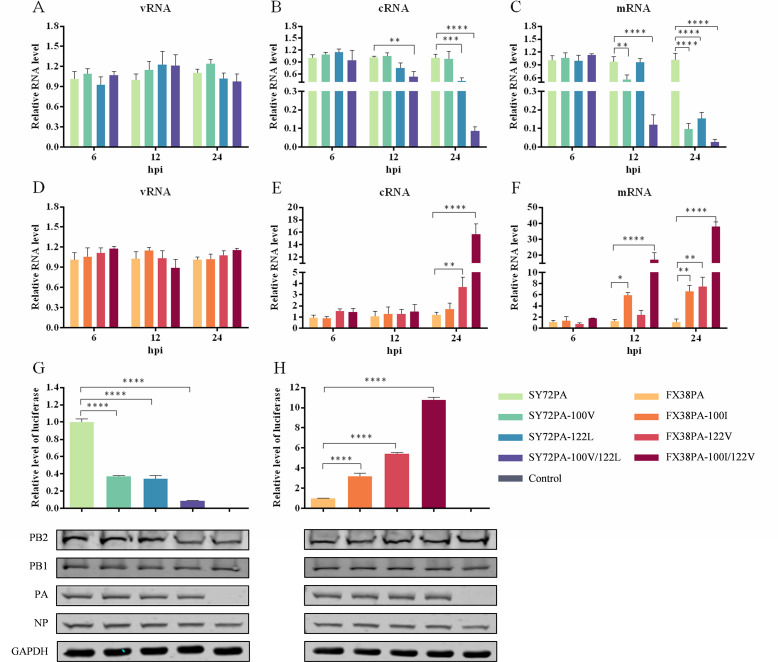
*In vivo* RNA synthesis mediated by the wild-type SY72-PA, FX38-PA, and their respective mutants. (A–C) The vRNA, cRNA, and mRNA levels of the luciferase gene mediated by SY72-PA, SY72-PA100V, SY72-PA122L, and SY72-PA100V/122L. (D–F) The vRNA, cRNA, and mRNA levels of the luciferase gene mediated by FX38-PA, FX38-PA100I, FX38-PA122V, and FX38-PA100I/122V. (**G and H**) Relative luciferase activity of the wild-type SY72-PA, FX38-PA, and their respective mutants. The recombinant plasmid pPolI-vRNA was constructed by inserting the luciferase gene bearing the conserved 5*'* and 3*'* end sequences of influenza virus RNA into the pPolI plasmid to evaluate the vRNA, cRNA, and mRNA levels in cells co-transfected with different vRNP plasmids. Levels of vRNA, cRNA, and mRNA were quantified by RT-qPCR, and the relative luciferase activity was determined with a GloMax 96 microplate luminometer. Data shown are means ± SD of three independent experiments, normalized to the mRNA of GAPDH. *P < 0.05; **P < 0.01; ***P < 0.001; ****P < 0.0001.

Comprehensive studies have shown that influenza virus transcription is carried out through the process of vRNA into messenger RNA (mRNA), while virus replication involves the processes of vRNA into complementary RNA (cRNA) and cRNA into vRNA (34, 35). To investigate the role of the two amino acid residues 100 and 122 in the PA protein in viral transcription and replication, a vRNA-encoding plasmid, designated as pPolI-vRNA, was constructed by inserting the full-length sequence of the luciferase reporter gene with the upstream 5*′* noncoding sequence and the downstream 3′ noncoding sequence of the NP gene. HEK293T cells were co-transfected with pPolI-vRNA, pCAGGS-SY72PB2, pCAGGS-SY72PB1, pCAGGS-SY72NP, and parental pCAGGS-SY72PA or mutated pCAGGS-SY72PA100V, pCAGGS-SY72PA122L, and pCAGGS-SY72PA100V/122L. Cell lysates were collected at 6, 12, and 24 hours post-transfection (hpt), and the vRNA, cRNA, and mRNA levels of the luciferase gene were determined by reverse transcription-quantitative polymerase chain reaction (RT-qPCR) as described previously (10). No significant difference in the vRNA level was detected between cells transfected with the parental pCAGGS-SY72PA and mutated pCAGGS-SY72PA100V, pCAGGS-SY72PA122L, or pCAGGS-SY72PA100V/122L at three timepoints (Fig. 7A). The cRNA levels of cells transfected with pCAGGS-SY72PA122L and pCAGGS-SY72PA100V/122L were 2.4- and 11.8-fold lower than cells transfected with pCAGGS-SY72PA, respectively, at 24 hpt (Fig. 7B). Compared to cells transfected with pCAGGS-SY72PA, the mRNA levels of cells transfected with pCAGGS-SY72PA100V, pCAGGS-SY72PA122L, and pCAGGS-SY72PA100V/122L were decreased by 10.5-, 6.7-, and 37.1-fold, respectively, at 24 hpt (Fig. 7C). Similarly, HEK293T cells were co-transfected with pPolI-vRNA, pCAGGS-FX38PB2, pCAGGS-FX38PB1, pCAGGS-FX38NP, and pCAGGS-FX38PA, or pCAGGS-FX38PA100I, pCAGGS-FX38PA122V, and pCAGGS-FX38PA100I/122V. There were no significant differences in the vRNA levels between the parental group and the three mutant groups (Fig. 7D). The cRNA levels of cells transfected with pCAGGS-FX38PA122V and pCAGGS-FX38PA100I/122V were enhanced by 3.1- and 13.0-fold, respectively, compared to cells transfected with pCAGGS-FX38PA (Fig. 7E). The mRNA levels of cells transfected with pCAGGS-FX38PA100I, pCAGGS-FX38PA122V, and pCAGGS-FX38PA100I/122V were increased by 6.0-, 6.8-, and 34.9-fold, respectively, compared to the parental group (Fig. 7F). In addition, the relative luciferase activity of cells transfected with plasmids containing pCAGGS-SY72PA100V, pCAGGS-SY72PA122L, and pCAGGS-SY72PA100V/122L was 2.67-, 2.93-, and 11.6-fold lower than that of cells transfected with pCAGGS-SY72PA at 24 hpt, respectively (Fig. 7G), while luciferase activity of cells transfected with pCAGGS-FX38PA100I, pCAGGS-FX38PA122V, and pCAGGS-FX38PA100I/122V was 3.21-, 5.45-, and 10.75-fold higher than that of cells transfected with pCAGGS-FX38PA at 24 hpt, respectively (Fig. 7H). These results demonstrate that a single amino acid mutation at position 100 in PA impacts transcription of vRNA, a mutation at position 122 affects synthesis of cRNA and mRNA, and these two mutations have a synergistic effect in the virus life cycle.

### The amino acid residues 100 and 122 in PA are involved in viral transcription and replication by a different mechanism

**Fig 8:**
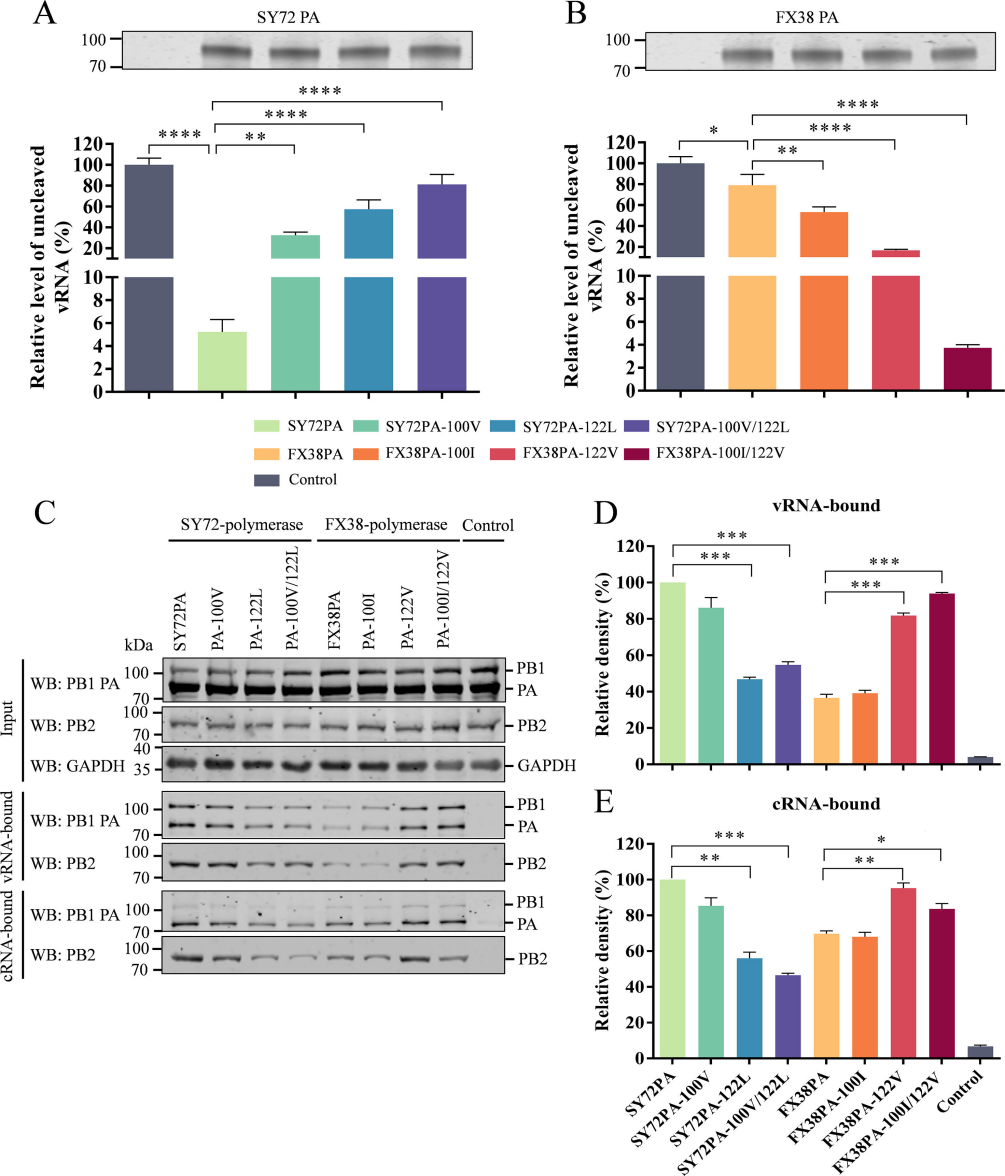
Mechanisms by which the amino acid residues 100 and 122 in PA plays a role in viral transcription and replication. (**A**) Endonuclease activities of the wild-type SY72PA, SY72PA-100V, SY72PA-122L, and SY72PA-100V/122L proteins. (**B**) Endonuclease activities of the wild-type FX38PA, FX38PA-100I, FX38PA-122V, and FX38PA-100I/122V proteins. Model vRNA was incubated with the wild-type or mutated PA protein in reaction buffer (20 mM Tris-HCl, 100 mM NaCl, 10 mM β-mercaptoethanol, and 1 mM MnCl_2_), and the uncleaved vRNA was quantified using RT-qPCR. Values in (**A) and (B**) are normalized to that of the control group (lane 1). (**C–E**) The polymerases containing wild-type and mutated PA protein pulled down by the model vRNA and cRNA. The interaction of polymerase–RNA complexes was assessed using western blot, and the density of the polymerase pulled-down by vRNA and cRNA was determined using ImageJ software (https://imagej.net/ij/). The wild-type SY72-polymerase pulled-down by the model RNA was set at 100% and that pulled-down by a non-targeted RNA was used as a negative control. Data shown are means ± SD of three experiments, *P* values compared to wild-type PA or polymerase. *P < 0.05; **P < 0.01; ***P < 0.001; ****P < 0.0001.

The N-terminus of the PA protein contains an endonuclease domain responsible for cleavage of the host mRNA during cap snatching and transcription (36, 37). The amino acid residues at positions 100 and 122 in PA identified in this study are located in the endonuclease domain of the PA protein. Herein, we determined the endonuclease activities of the different PA proteins purified from the transfected HEK293T cells with pCAGGS-SY72PA, pCAGGS-SY72PA100V, pCAGGS-SY72PA122L, and pCAGGS-SY72PA100V/122L, respectively. The model vRNA was obtained *in vitro* as previously described (10) and incubated with a similar amount of PA protein at 37°C for 40 min. Then, the model vRNA that was not cleaved in the reaction was quantified by RT-qPCR. As shown in Fig. 8A, the percentage of the uncleaved model vRNA increased to 32.6% and 57.5% after incubation with SY72PA-100V and SY72PA-122L, respectively, compared with that after incubation with SY72PA, which was 5.23%. Especially, the percentage of the uncleaved model vRNA dramatically increased to 81.2% after incubation with SY72PA-100V/122L. We did a similar test with the FX38PA, FX38-PA100I, FX38-PA122V, and FX38PA-100I/122V proteins and found that 79.0% of the model vRNA remained uncleaved after incubation with FX38PA, whereas 53.3%, 16.7%, and 3.7% of the model vRNA was intact after incubation with FX38PA-100I, FX38PA-122V, and FX38PA-100I/122V, respectively (Fig. 8B). These data indicate that the mutations I100V and V122L in the PA of SY72 reduce its endonuclease cleavage activity, while the mutations V100I and L122V in the PA of FX38 enhance its endonuclease cleavage activity.

Functional studies have shown that the N-terminal domain of PA contains regions involved in binding to the vRNA/cRNA promoter (38). To determine the effects of the two amino acid mutations at positions 100 and 122 of PA on its ability to bind RNA, we measured the interactions between the polymerase complex and the vRNA/cRNA promoter by an RNA-protein pull-down assay. As shown in Fig. 8C, the polymerase complexes of SY72 and FX38 were prepared from the HEK-293T cells co-transfected with plasmids expressing PB2, PB1, and wild-type or mutant PA and further characterized using western blot. After incubation of the polymerase complexes with the model RNA, the amounts of RNA-bound protein was measured by infrared imaging and its quantitative evaluation. As shown in Fig. 8D, the vRNA-bound protein amounts of the polymerase carrying SY72PA-100V SY72PA-122L and SY72PA-100V/122L were 2.1- and 1.8-fold smaller than that of the wild-type SY72-polymerase, respectively. However, there was no difference in the protein amounts of the polymerase harboring SY72PA-100V and SY72PA. Meanwhile, the vRNA-bound protein amounts of the polymerase carrying FX38PA-122V and FX38PA-100I/122V were 2.2- and 2.6- fold greater than that of the FX38-polymerase, respectively. However, the protein amount of the polymerase harboring FX38PA-100I was comparable to that of the FX38-polymerase. We further investigated the binding of polymerase to cRNA and found that the cRNA-bound protein amounts of the polymerase containing SY72PA-122L and SY72PA-100V/122L were 1.8- and 2.1- fold smaller than that of the SY72 polymerase, whereas the protein amounts of the polymerase containing FX38PA-122V and FX38PA-100I/122V were 1.4- and 1.2- fold greater than that of the FX38-polymerase, respectively (Fig. 8E). These results indicate that a single amino acid mutation at position 122 in PA affects the ability of the polymerase to bind vRNA and cRNA, thereby, plays an important role in viral replication.

### Polymorphism analysis of amino acids at positions 100 and 122 of the H1N1 PA protein

To investigate the polymorphism of the amino acids residues 100 and 122 in PA of the H1N1 viruses, we downloaded the PA sequences of H1N1 viruses respective of host origins and lineages from the NCBI Influenza Virus Resource (https://www.ncbi.nlm.nih.gov/genomes/FLU/Database/nph-select.cgi?go=database; up to 26 November 2023) and the Bacterial and Viral Bioinformatics Resource Center (https://www.fludb.org/brc/home.spg?decorator=influenza; up to 26 November 2023). Alignments of the amino acid sequences of the corresponding regions of the PA proteins were conducted using the MAFFT (Multiple Alignment using Fast Fourier Transform) program (https://mafft.cbrc.jp/alignment/software/). As shown in Table 1, among the human-origin H1N1/2009 viruses isolated from 2009 to 2023, PA-100V was the dominant during 2009 and 2012, then it was gradually replaced by PA-100I. Of note, PA-100V was not found in the human-origin H1N1/2009 viruses isolated from 2021 to 2023. Similarly, PA-100V was gradually replaced by PA-100I in the swine-origin H1N1/2009 viruses isolated from 2009 to 2023, with the percentage of PA-100I increasing from 0.4% to 46.2%. We further analyzed the H1N1/2009 PA gene denoted in the rEA H1N1 viruses and found that PA-100V dominated among the swine- and human-origin viruses isolated during 2009 and 2023. In addition, PA-100V dominated in the classical swine H1N1, pure EA H1N1, and avian H1N1 influenza viruses, while PA-100A dominated in the human seasonal H1N1 influenza viruses. Interestingly, PA-122V was highly conserved, while PA-122L was not detected among the H1N1 viruses analyzed in this study, with the exception of the FX38 virus isolated in our surveillance activity.

**TABLE 1:** Polymorphism frequencies of the amino acid residues 100 and 122 in PA of H1N1 influenza viruses^*a*^

Host and H1 clade (n)	Amino acid residue 100	Prevalence (%) of the indicated amino acid at different time periods					Amino acid residue 122	Prevalence (%) of the indicated amino acid at different time periods				
		Pre-	2009–2012	2013–2016	2017–2020	2021–2023		Pre-	2009–2012	2013–2016	2017–2020	2021–2023
Human												
H1N1/2009 (14,277)	Val		99.2 (5,095)	12.7 (327)	0.2 (8)	0	Val		99.9 (5,136)	99.9 (2,566)	99.9 (4,994)	100 (1,572)
	Ile		0.6 (33)	86.8 (2,231)	98.5 (4,925)	99.6 (1,566)	Leu		0	0	0	0
rEA H1N1 (4)^*b*^	Val			100 (2)	100 (2)		Val			100 (2)	100 (2)	
EA H1N1 (10)	Val	100 (6)	100 (2)	100 (2)			Val	100 (6)	100 (2)	100 (2)		
Seasonal H1N1 (1,008)^*c*^	Val	2.7(25)	3.6 (3)				Val	99.8 (922)	100 (83)			
	Ile	0	0				Leu	0	0			
Swine												
H1N1/2009 (1,259)	Val		99.4 (478)	84.9 (383)	59.6 (187)	30.8 (4)	Val		100 (481)	99.8 (450)	100 (314)	100 (13)
	Ile		0.4 (2)	12.9 (58)	39.5 (124)	46.2 (6)	Leu		0	0	0	0
rEA H1N1 (377)^*b*^	Val		100 (28)	95.0 (208)	85.9 (85)	93.5 (29)	Val		96.4 (27)	99.1 (217)	99.0 (98)	100 (31)
	Ile		0	4.1 (9)	13.1 (13)	6.5 (2)	Leu		0	0	1.0 (1)	0
EA H1N1 (491)	Val	96.7 (117)	96.4 (266)	96.7 (88)	100 (3)		Val	100 (121)	98.9 (273)	100 (91)	100 (3)	
	Ile	3.3 (4)	3.3 (9)	2.2 (2)	0		Leu	0	0	0	0	
Classical H1N1(1,850)	Val	98.0 (393)	98.1 (528)	85.8 (399)	78.3 (349)		Val	100 (401)	99.6 (536)	99.6 (461)	100 (446)	
	Ile	1.8 (7)	1.5 (8)	10.5 (49)	21.7 (97)		Leu	0	0	0	0	
Avian												
H1N1 (660)	Val	99.3 (274)	100 (165)	93.9 (139)	93.4 (57)	100 (10)	Val	99.6 (275)	100 (165)	100 (148)	100 (61)	100 (10)
	Ile	0.4 (1)	0	6.1 (9)	3.3 (2)	0	Leu	0	0	0	0	0

^*a*^ The amino acid frequencies were determined for unique PA full-length sequences downloaded from the NCBI Influenza Virus Resource and Bacterial and Viral Bioinformatics Resource Center. n, number of sequences analyzed.

^*b*^ “rEA H1N1 viruses” refers to viruses carrying the PA gene of H1N1/2009 viruses.

^*c*^ Majority of human seasonal H1N1 viruses carry PA-100A (Ala), with the percentages of 97.3% pre-2009 and 96.4% during 2009–2012, respectively.

### A single V100A or V100I substitution in the PA protein enhances polymerase activity of the human-origin rEA H1N1 virus

**Fig 9:**
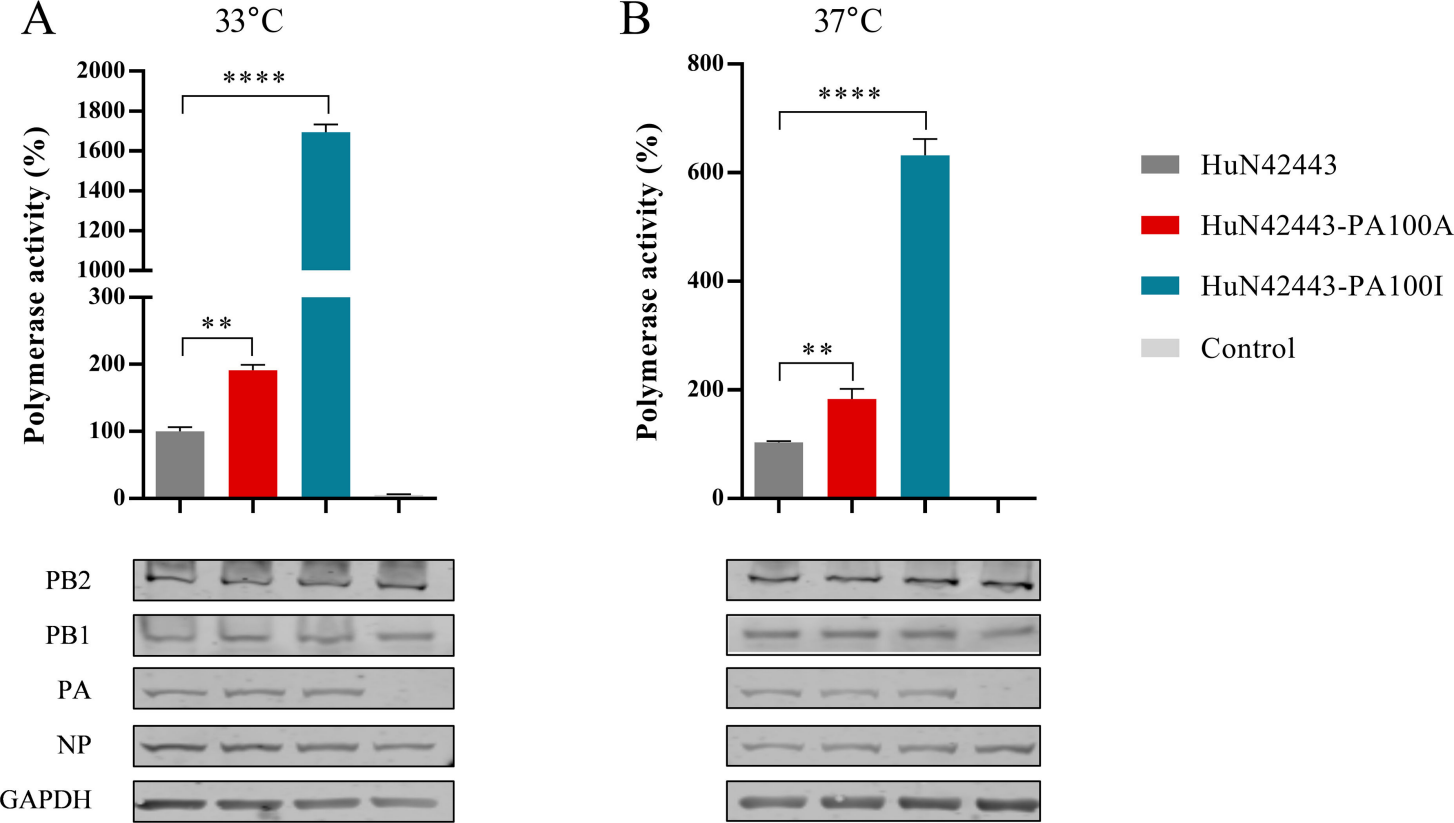
Effects of a single amino acid substitution at position 100 in the PA protein on polymerase activity of the reconstituted vRNP. (**A and B**) Polymerase activities of the reconstituted vRNP at 33°C and 37°C containing a single amino mutation V100A and V100I in PA were determined as described in the legend for Fig. 4. *P* values compared to wild-type vRNP. **P < 0.01; ****P < 0.0001.

To investigate the biological function of the amino acid residue 100 in PA of the other viruses, we introduced the mutation V100I or V100A into the PA gene of a human-origin rEA H1N1 virus (A/Hunan/42443/2015 [HuN42443]), which showed high virulence in C57BL/6 J mice (29). Then, we tested the polymerase activities of the wild-type vRNP and reconstituted vRNP containing mutant PA protein of the HuN42443 virus. As shown in Fig. 9A and B, the polymerase activities of the reconstituted vRNP bearing HuN42443-PA100A and HuN42443-PA100I were increased by 1.9- and 16.9- fold at 33°C, and by 1.8- and 6.1- fold at 37°C, respectively, compared with that of the vRNP of HuN42443.

## DISCUSSION

Although the rEA H1N1 viruses do not cause severe disease in pigs, they exhibited enhanced virulence and effective transmission abilities in mammalian models after acquiring the internal genes of H1N1/2009 viruses (10, 28, 39). The possibility of zoonotic transmission and emergence of new pathogenic influenza strains of pandemic potential poses a significant public health threat. In this study, we demonstrated that two naturally isolated viruses, FX38 and SY72, carry the HA, NA, and M genes of EA H1N1 viruses, the PB2, PB1, PA, and NP genes of H1N1/2009 viruses, and the NS gene of triple-reassortment H1N2 viruses. The two viruses differed in the virulence in BALB/c mice. The SY72 virus replicated with higher titers in the nasal turbinates and lungs, and induced apparent body weight loss of mice with an MLD_50_ of 3.32 log_10_ EID_50_. However, the FX38 virus replicated with lower titers in the organs of mice and induced slight body weight loss, with an MLD_50_ ≥6.5 log_10_ EID_50_. We identified two amino acids at positions 100 and 122 in PA, which have individual and cumulative effects on the polymerase activity, viral replication capacity, and viral pathogenicity of EA H1N1 influenza viruses in mice. We further explored the roles of these two residues in viral transcription and replication, and found that amino acid residue 100 is involved in transcription of vRNA by altering the endonuclease activity of PA protein, and residue 122 is involved in the synthesis of both mRNA and cRNA by influencing the endonuclease activity and RNA-binding activity of PA protein.

The PA protein, a subunit of viral polymerase complex, is essential for viral replication, pathogenicity. and adaptation to new host species of influenza viruses (40–47). The N-terminal region of PA is involved in multiple functions of the polymerase, including protein stability, endonuclease activity, cap binding, and vRNA/cRNA promoter binding, playing important roles in initiating both transcription and replication through different mechanisms (48). Previous studies found that the first 247 amino acids in the N-terminal of PA preserved the ability to induce *in vivo* protein degradation (49, 50). In the present study, the amino acids at positions 100 and 122 are located in the N-terminal region of the PA protein. We introduced a single or double amino acid mutation at positions 100 and 122 in PA. No significant difference was detected in the expression level between the parental and mutant PA, which indicated that these two amino acids do not affect the stability of PA protein (data not shown). Furthermore, our data indicated that the amino acid mutation at position 100 in PA had a significant effect on the mRNA level, but no effect on the vRNA, or cRNA level, and verified the previous finding that amino acid residue 100 impacted on endonuclease activity of PA and therefore affected mRNA transcription (10). However, our results were inconsistent with other previous findings that the single A100V mutation showed no significant effect on the mRNA, vRNA, or cRNA levels between the mutant and wild-type PA of H1N1/1918 virus (48), and the single V100A mutation in PA was found to enhance transcription of vRNA, but reduce synthesis of cRNA, though it had no significant effect on the viral growth capacity and polymerase activity of the mutant in the A/Netherlands/219/2003 (H7N7) backbone (51). It is speculated that the function of amino acid residue 100 in different PA proteins is strain-dependent in the viral transcription or replication.

Adaptation is considered to drive evolution by conferring mutations that enhance fitness and natural selection (52). A previous study found that the avian PA genes have been introduced into swine populations multiple times and eventually seeded the PA gene of H1N1/2009 virus (53). Our and previous findings indicated that the amino acid mutation V100I in PA gradually increased during the transmission of H1N1/2009 viruses in humans and pigs, then introduced into the rEA H1N1 viruses (10). Of note, we found that PA-122V was highly conserved in the H1N1 influenza viruses, suggesting an important role of this amino acid in the polymerase complex, though there is insufficient data on this residue in relation to virulence in mice. A previous study reported that V122T mutation had no effect on the polymerase activity (54). In this study, we first clarified the individual and synergistic effects of amino acid residue 122 in PA on the polymerase activity, viral replication, and virulence in mice. To our knowledge, the residue 122 in the PA protein has not been previously characterized or implicated in the evolution of influenza viruses. Although its source is not known, PA-122L possibly resulted from interactions between the different subunits of the influenza virus polymerase complex and various host cell proteins. Our results add to the growing body of mutational analysis data defining the function of the PA subunit and potential roles in adaptation to mammalian hosts.

A previous study revealed that there were several amino acids in PA distinguishing human and avian influenza polymerases, in which human isolates had 100A, whereas avian isolates had 100V (55). The PA protein of H1N1/2009 virus was avian-like genetic character, which harbored seven unique avian virus residues (28P, 55D, 57R, 65S, 100V, 312K, and 552T) and three human virus residues (356R, 382D, and 409N) (56). In the present study, the PA genes of FX38 and SY72 viruses were originated from H1N1/2009 virus. Among eight different amino acids in PA of these two viruses, a unique amino acid at position 100 in PA of the SY72 virus mutated from avian-like 100V to 100I, which is a molecular determinant involved in the enhanced polymerase activity. We also found that the human-origin rEA H1N1 viruses carried 100V, rather than 100A or 100I, in PA. Considering the great significance of the amino acid residue 100 in host adaptation, we further revealed the enhanced effect of 100A or 100I on the polymerase activity. Notably, the V100I mutation dramatically enhanced the polymerase activity by 16.9-fold at 33°C, which is reported to be closely correlated with the viral replication ability in the upper respiratory tract and thereby maybe increase the viral transmissibility.

Efficient transmission via respiratory droplets is one of the prerequisites for influenza virus to cause a pandemic, and ferrets have been widely used as model animals for evaluating the transmissibility of influenza viruses. The findings of our previous studies and others indicated that some of the rEA H1N1 viruses bearing the internal genes of H1N1/2009 virus exhibit efficient infectivity and transmissibility in ferrets (10, 27, 28) and further found that four amino acids (100I, 330V, 321K, and 639T) in PA play a key role in the transmissibility of the rEA H1N1 viruses in ferrets (10). In this study, we identified two residues 100 and 122 in PA that are crucial determinants of virulence of rEA H1N1 viruses in a murine model and revealed the individual and synergistic effects of these two residues on the polymerase activity and viral replication capacity *in vitro*. Our findings into the effects of the two residues on viral transcription and replication give new insights for better understanding of the molecular mechanisms of the PA protein determining the virulence of influenza viruses. However, the specific roles of these two residues of PA in virulence in pigs and transmissibility of rEA H1N1 viruses remains to be further investigated.

## MATERIALS AND METHODS

### Cells and viruses

Madin–Darby canine kidney (MDCK) and human embryonic kidney (HEK293T) cells were maintained in Dulbecco’s modified Eagle’s medium (DMEM) (Gibco, Grand Island, NY, USA) supplemented with 5% and 10% fetal bovine serum (Gibco), respectively. Two H1N1 SIVs, FX38 and SY72, were isolated from the nasal swabs collected from pigs in slaughter houses located in Liaoning province, China, during 2017 and 2018. All the viruses were propagated in 10-day-old specific pathogen-free embryonated chicken eggs, followed by determining the EID_50_.

### Genomic sequencing and phylogenetic analyses of the viruses

The whole-genome sequencing of the FX38 and SY72 viruses were performed as described previously (26). For phylogenetic analysis, the sequences of 79 H1N1 reference strains were downloaded from the Bacterial and Viral Bioinformatics Resource Center (https://www.fludb.org/brc/home.spg?decorator=influenza; up to 26 November 2023). The phylogenetic tree of the isolates was generated by the distance-based neighbor-joining method using the software MEGA7.0.26 (www.megasoftware.net). Reliability of the tree was assessed by bootstrap analysis with 1,000 replicates. Horizontal distance is proportional to genetic distance.

### Rescue of reassortant viruses

Reverse genetic systems for the FX38 and SY72 viruses were established by inserting the full-length cDNA derived from the respective virus into the vRNA–mRNA bidirectional transcription vector pHW2000 as described previously (26). Then, a series of reassortants were generated by single-gene substitution and site-directed mutagenesis. Recombinant viruses were rescued by transfection in HEK293T cells and identified by whole-genome sequencing to ensure the absence of unwanted mutations. The sequences of primers for virus rescue are available upon request.

### Virus growth kinetics

To evaluate viral replication properties *in vitro*, confluent monolayers of MDCK cells were infected with the indicated viruses at a multiplicity of infection (MOI) of 0.001. After incubation for 1 h at 37°C, the viral inoculum was replaced with DMEM containing 1 µg/mL of tolylsulfonyl phenylalanyl chloromethyl ketone-treated trypsin (Sigma-Aldrich, St. Louis, MO, USA), followed by further incubation at 37°C. Cell culture supernatants were collected at 12, 24, 36, 48, 60, and 72 hpi for virus titration in MDCK cells.

### Minigenome assay

A minigenome assay was performed to determine viral polymerase activity. Briefly, HEK293T cells were transfected with pcDNA3.1(+) plasmids encoding PB2, PB1, NP, and PA (the wild type or mutant), together with a plasmid expressing a ﬁreﬂy luciferase gene under the control of RNA polymerase I promoter (pPolI-Luc), and pRL-TK (Promega, Madison, WI, USA), which expresses *Renilla* luciferase, as an internal control. The ﬁreﬂy and *Renilla* luciferase activities were measured using the Dual-Luciferase Reporter Assay System (Promega, Madison, WI, USA). Polymerase activity was calculated by standardization of the ﬁreﬂy luciferase activity to the *Renilla* luciferase activity. Polymerase activities of the wild-type vRNP of the FX38 and SY72 viruses were set at 100%. Experiments were performed in triplicate.

### Pathogenicity study in mice

Six-week-old female BALB/c mice (Vital River Laboratories, Beijing, China) were used to determine the MLD_50_ value and replication capacities *in vivo* of all viruses. Groups of five mice were anesthetized with averdin (Sigma-Aldrich) and intranasally inoculated with 10^2^ to 10^6^ EID_50_ of the indicated viruses in 50 µL of phosphate-buffered saline (PBS). Body weight and survival were monitored daily for 14 dpi. Mortality was recorded either as an actual death or body weight loss ≥25%, which is the threshold for humane euthanasia. To evaluate the viral replication capacity *in vivo*, three mice were euthanized at 3 dpi from each group inoculated with 10^6^ EID_50_ of virus. The organs, including the brain, nasal turbinate, lungs, spleen, and kidneys, were harvested and titrated in 10-day-old embryonated chicken eggs.

### RT-qPCR of vRNA, cRNA, and mRNA

HEK293T cells were transfected with pCAGGS-PB1, pCAGGS-PB2, pCAGGS-PA (the wild type or mutant), pCAGGS-NP, and pPolI-vRNA plasmids using Lipofectamine LTX Reagent with PLUS Reagent (Thermo Fisher Scientific, Waltham, MA, USA). Total RNA was extracted from cells harvested at 6, 12, and 24 hpt using RNAsimple Total RNA Kit (Tiangen, Beijing, China). The corresponding cDNAs of vRNA, cRNA, and mRNA were transcribed with respective tagged primers using the HiScript II 1st Strand cDNA Synthesis Kit (Vazyme, Nanjing, China), then subjected to qPCR with 2× SYBR green PCR master mix (Vazyme), according to the manufacturer’s instructions. The primer sequences for RT and qPCR of vRNA, cRNA, and mRNA were referred as described previously (10). The expression level of each gene was normalized to expression of glyceraldehyde 3-phosphate dehydrogenase (GAPDH) as a control using the 2^−ΔΔCT^ method. Each experiment was performed in triplicate.

### Endonuclease assay

Endonuclease assay was carried out as previously described (10). Briefly, a partially folded panhandle RNA of 300 nucleotides consisting of the conserved 5′ and 3′ ends of the NP gene was generated *in vitro* using a MEGAscript T7 Transcription Kit (Thermo Fisher Scientific) and designated model vRNA. Cleavage of the model vRNA was determined by incubation of 10 µM PA protein with 500 ng of panhandle RNA for 40 min at 37°C in reaction buffer containing 20 mM Tris-HCl, 100 mM NaCl, 10 mM β-mercaptoethanol, and 1 mM MnCl_2_. The incubation was stopped by the addition of 20 mM egtazic acid. RNA products were recovered using a Spin Column RNA Cleanup and Concentration Kit (Sangon Biotech Co. Ltd., Shanghai, China) and quantified by RT-qPCR.

### RNA-protein pull-down assay

Model RNA was prepared as previously described in endonuclease assay and labeled using a Pierce RNA 3′ End Desthiobiotinylation Kit (Thermo Fisher Scientific). The polymerase trimers were prepared from the HEK293T cells co-transfected with pCAGGS-PB2, pCAGGS-PB1, and pCAGGS-PA (the wild type or mutant) and incubated with the labeled RNA using a Pierce Magnetic RNA-Protein Pull-Down Kit (Thermo Fisher Scientific). RNA-binding protein complexes were precipitated on a magnetic stand and eluted with elution buffer. The amount of protein was analyzed using western blot.

### Western blot analysis

Protein samples were separated by SDS-PAGE and transferred onto nitrocellulose membranes (GE Healthcare Life Sciences, Chicago, IL, USA), then probed with monoclonal antibodies against target proteins (PB2, PB1, PA, NP, or GAPDH) and IRDye800CW goat anti-mouse IgG (H + L). Blots were visualized using an Odyssey Infrared Imaging System (LI-COR Biosciences, Lincoln, NE, USA).

### Statistical analysis

Statistical significance was determined using Student’s *t*-test for two-group comparisons and one-way or two-way analysis of variance with Tukey’s post-test for multiple-group comparisons in GraphPad Prism version 8.0 (GraphPad Software Inc., CA, USA). A probability (*P*) value <0.05 was considered statistically significant. Error bars indicate the SD of three independent experiments.

## Data Availability

The eight gene segments of the isolates were sequenced and have been deposited in GenBank under accession numbers OR984412-OR984419 and OR984426-OR984433.
